# Diseases of the Oral Mucous Membrane

**Published:** 1891-09

**Authors:** J. D. Patterson

**Affiliations:** Kansas City, Mo.


ARTICLE IV.

### DISEASES OF THE ORAL MUCOUS MEMBRANE.

BY J. D. PATTERSON, D. D. S., KANSAS CITY, MO.

Read before the American Dental Association, Saratoga Springs, N. Y., August 5, 1891.

Some of the diseases of the oral mucous membrane, their pathology, and their effect upon the dental organs,—the teeth,—will be the points considered in this article.

It behooves the dental profession to take cognization of the fact that this subject has been very much neglected, because its importance has been underrated. Until the agitation brought about by investigation of the disease called pyorrhea alveolaris, there had been little attention to diseases which were of the mucous membrane *per se*. Since then investigators, although laboring under misconceptions, have recognized more than they did the importance to the intelligent dental practitioner of a thorough knowledge of the etiology and pathology of these complaints.

Much is still undecided, and the diffusion of such knowledge as may be said to have become generally accepted goes on very slowly.

The inception of the irritation by which we have disease of the oral mucous membrane may be divided into classes. It may be accidental, constitutional, or hereditary. Investigation and observation compels the belief that the active causes are seldom hereditary; they are often acquired conditions, but very largely due to the accidental in their inception, hereditary or constitutional factors merely acting as predisposing conditions.

I desire in this article to especially call attention to some of these accidental causes We find that they are very numerous. The irritation may be mechanical alone, may result from the invasion of micro-organisms, from lack of hygienic care, from disuse, from infection, or from functional disturbances without mechanical aid.

We will first consider the cause of disease in this membrane by any arrest or disturbance of its function. The dental profession have not given so much thought to this particular line of investigation as to the search after irritants found in calcareous irritation, to microbian theories, or constitutional conditions, and therefore, we believe, have signally failed to explain the etiology of certain symptoms often found in this membrane, especially in the early stages of pyorrhea, where no irritation, constitutional or active, could be found; the disease being established, passing through its different stages from irritation to suppuration and loss of tissue, and giving to the observer nothing of its etiology, except in theories unsupported by any clinical fact. Our purpose is to study this membrane and endeavor to point out where the obscure irritation arises.

In glancing at the anatomy of the oral mucous membrane, it may be briefly described as a layer of epithelium with a connective tissue framework, and glands underneath supporting the epithelial layer, and provided with blood-vessels, lymphatics and nerves. The glands are of the tubular character, and are imbedded in the connective-tissue framework. Their function is to produce mucus. In health, mucus of normal quantity and quality is constantly produced. It is of a transparent, watery color, diffused over the surface. It serves to keep the parts moist, pliable, and in a state of comfort, and protects from the irritating influence of external matters.

The epithelial layer is in a constant condition of growth of the deeper and desquamation of the superficial cells. Whatever interferes with and changes this normal and physiological condition and function in this membrane may overcome its power of resistance, and disease will result. The oral mucous membrane is especially liable to changes in temperature, which interfere with its health. The effect of lower temperature, often met with in external causes, will be found to be a chilling of this membrane, which contracts its epithelium until the mucous ducts are prevented from pouring out their secretion, and functional derangement is thus established. This is commonly termed "taking cold." Any marked deviation from the normal heat of the tissues, viz., 98° F., may result in morbid changes.

Heat-production is going on all over the body. When any of the tissues are subjected to cold, this heat-production is stopped and morbid action comes. In the mouth the contraction of the mucous membrane is the first step, accompanied by stoppage of the mucous secretion. Quickly succeeding this we have increased nutrition, an over-production of the mucous secretion on account of increased blood-supply, which is the result of irritation from disturbed function; a slightly reddened tissue follows from the same cause, and an abundant effusion of serum escapes; the epithelium cells fall off more rapidly than under normal conditions. As the inflammation progresses, and stasis and the other classic signs of inflammation ensue, we see the mucous discharge rapidly becoming purulent, because so many unripe cells quickly become pus cells. Micro-organisms now make their appearance, and assist in the advanced stages of the disease. This process soon extends below the epithelium, and the membrane, becoming infiltrated with depraved cell-elements, swells and thickens, the inflammatory products resulting in suppuration in convenient territory.

Let it be borne in mind that until the advanced stage in the conditions described, there is not of necessity any presence of pathogenic or non-pathogenic micro-organisms. The irritation in its inception has been simply through an arrest of function. Neither have there been of necessity any hereditary or constitutional favoring conditions, without which these results would not have been manifest. Favorable hereditary condition of tissue and constitutional environment are in these cases merely predisposing factors. In other diseases of the mucous membrane exhibiting similar symptoms it is not to be doubted that constitutional conditions and peculiar diatheses figure largely, and that under such conditions and with the presence of pathogenic micro-organisms the mucous membrane is involved even at the inception of disease; but there are greater factors which explain some symptoms without seeking in the field of bodily conditions, micro-organisms and mechanical irritation.

I pass now to another irritant of the oral mucous membrane, viz: the prevalent habit of "mouth-breathing." Leaving out of the question the change in the temperature of the mouth by this habit, we find that respired as well as inspired air rapidly dries the surface of the mucous membranes destroying the moistened lubricating surface, stopping the mucous ducts until the same train of symptoms from disturbed function already described is established. When a subject presents, who from one cause or another, breathes through the mouth, the intelligent operator can certainly predict the condition of the oral mucous membrane. It will be found in some of the varied stages of inflammation, acute or chronic, modified in event by the degree of hygienic care bestowed by the patient.

Here we have again a numerous class of cases where undeniably the disease has arisen from the simple drying of the naturally moist, lubricated surface by the introduction of an atmosphere which is not normal to the tissue. No micro-organisms need be present, nor has calcareous or other irritant matter or bodily predisposition anything to do with the real inception of the baleful line of results.

The condition described as arising from interference with the function of the oral mucous membrane is not entirely and exclusively pathognomonic of irritation from mouth-breathing, or shock, or the result of thermal change. These symptoms may result from many different causes. They are seen in infection from catarrhal, croupous, diphtheritic, or tubercular surroundings. They are superinduced also by medicinal treatment, by mechanical irritants, and by uncleanliness.

In this last cause is found the etiology of by far the greatest number of diseases of the oral mucous membrane. We find, however, that while the cause of the described conditions may be varied, and while the cause will measurably affect the malignancy of the disease, yet the pathological symptoms are very similar, and we are brought in contact with a *condition* which follows from *numerous* active causes.

This *condition* is a disturbed function. A physiological protest arises, profuse discharges ensue, inflammatory processes are established, which end in suppuration. The same clinical aspects are found in the nasal tract, in the pharynx, and all connecting cavities of the upper air tract. The chief symptom is an abnormal secretion or discharge of mucus or muco-pus, and—I care not that objection has been made by some authors to the application of the term "catarrh" to a lesion in the oral cavity, yet—a catarrh it is, just as inevitably as any fact established in pathological conditions. I repeat, however, that catarrh is merely a condition or symptom, and is present in many differently-caused diseases affecting the oral cavity and the upper air tracts.

In the oral cavity the train cf symptoms we have described meets with conditions which rapidly determine and further the inflammatory processes. The teeth are surrounded by mucous membrane, whose union with the teeth forms a somewhat abrupt break in its smoothness and continuity, affording a nidus for irritating matter to lodge and ferment. The gingival margins, it is weil known, rapidly become the focal points of disease. At this margin, then, we find the most noticeable results of the loss of function in the mucous membrane. Through continued inflammatory action we soon have destruction of the normal union of the tissue to the tooth, and then ensues the train of pathological conditions so well known and often described as pyorrhea alveolaris; which is simply a stage in the disease naturally resulting on account of the peculiar anatomy of the region, a sequence of preceding pathological disturbances, easily traced, and in favorable conditions surely following.

In speaking of diseases of this mucous membrane of the mouth resulting in what is familiarly known as pyorrhea alveolaris, different authors have expressed themselves strongly opposed to the use of the term catarrhal in describing the clinical appearance. They take this position because they are laboring under an entire misconception in regard to the term. They consider catarrh a *special*, *peculiar* disease of certain membranes which ultimately leads to ulceration and necrosis, when it is *not this at all*; it is simply a *condition*,—a condition of discharge,—and not a disease of special cause or pathology.

Another somewhat prevalent idea entertained by the medical as well as the dental profession is, that there is a catarrhal *diathesis*, a peculiar systemic condition, under which a patient speedily becomes liable to catarrhal inflammation. In regard to this, the celebrated Dr. F. H. Bosworth says, "I know of no such condition, and there is no good ground for the assertion. My clinical experience also fails to justify the view in any manner" (p. 101, "Diseases of the Throat and Nose". The fact is that the best clinical experience and observation of specialists in diseases of the mucous membrane agree that the conditions of the mucous membrane here described are, with few exceptions, of local origin; that the constitutional disturbance is only secondary, and that for the most part only local treatment is demanded.

Another argument against classifying these diseases of the oral mucous membrane as catarrhal comes from those who lay great stress upon climate influence. They find in climates where catarrhs are almost unknown many cases of pyorrhea, and, on the contrary, where catarrhs are endemic find no increase in the number of those suffering from discharges from the oral mucous membrane.

In regard to this, I think it must be considered a very loose statement, for, in the first place, statistics in the premises gathered by those who have devoted their lives to the treatment of diseases of the mucous membrane prove that those diseases are not peculiar to any climate or country. It was formerly supposed that these diseases were more prevalent in America than in Europe, but this has been disproved. It was found that they had received more attention from specialists in America at an early date, and so catarrh was called an American disease. It has been found, however, that it is equally prevalent in Europe.

There can be no question that a change of climate works wonders in catarrhal troubles once they are established, "but that climatic influence is an important factor in its causation is open to question" (Bosworth). The highest authorities agree upon this point.

In leaving the question for your discussion, I suggest the following considerations :

1. That the inception of disease in the oral mucous membrane, where the cause is obscure, is often to be found in the arrest of function in that membrane by thermal shock, by "catching cold," or by mouth-breathing, by which the moist, lubricated membrane is destroyed, rather than by hereditary conditions, constitutional diathesis, or the invasion of micro-organisms.

2. That the irritated mucous membrane which we find weeping out an abnormal quantity of mucus or mucopus is not pathognomonic of any special irritant, but is simply a condition.

3. That this condition is properly termed "catarrh."

4. That catarrh is not a special disease, but is merely a *condition* of disease.

5. That the name pyorrhea alveolaris simply denotes a stage in catarrh of the oral mucous membrane, is not pathognomonic, but may result from a great variety of causes.—*Dental Cosmos.*

